# The effects of virtual reality and video conferencing on personal and relational well-being among older adults with cognitive impairments and their adult children: The Thrive randomized controlled trial

**DOI:** 10.21203/rs.3.rs-10584186/v1

**Published:** 2026-08-27

**Authors:** Tamara D. Afifi, Nancy L. Collins, Kyle Rand, Jennifer J. Stamps, Erin E. Naffziger, Nikki Truscelli, Paige E. Harris, Thomas Neumann, Allison P. Mazur, Yuval Rosen, Abdullah S. Salehuddin, Chloe Gonzales, Christopher D. Otmar, Veronica Wilson, Jade Salmon, Ken K. Fujiwara, Joan K. Monin, Yifan Lou

**Affiliations:** Department of Communication, University of California, Santa Barbara, CA, USA; Department of Psychological and Brain Sciences, University of California, Santa Barbara, CA, USA; Rendever, Boston, MA, USA; Rendever, Boston, MA, USA; Rendever, Boston, MA, USA; Department of Communication, University of California, Santa Barbara, CA, USA; Department of Psychological and Brain Sciences, University of California, Santa Barbara, CA, USA; Rendever, Boston, MA, USA; Department of Communication, University of California, Santa Barbara, CA, USA; Rendever, Boston, MA, USA; Department of Communication, University of California, Santa Barbara, CA, USA; Department of Communication, University of California, Santa Barbara, CA, USA; Department of Communication, University of California, Santa Barbara, CA, USA; Department of Communication, University of California, Santa Barbara, CA, USA; Department of Communication, University of California, Santa Barbara, CA, USA; Department of Psychology, National Chung Cheng University, Chia-Yi Taiwan (R.O.C.); Yale School of Public Health, Yale University, New Haven CT, USA; School of Social Work, Virginia Commonwealth University, Richmond, VA, USA

## Abstract

Older adults in senior living communities (SLCs) often face barriers to maintaining emotional well-being and family ties. This stratified, unblinded, parallel-group randomized controlled trial tested a four-week virtual reality (VR) intervention (*Thrive*) designed to improve well-being for older adults with cognitive impairment and their adult children living at a distance, compared to an active video conferencing (VC) control. A total of 186 dyads – each comprising a parent with mild cognitive impairment (MCI) or mild-to-moderate Alzheimer’s disease and related dementias (ADRD) and an adult child – were recruited from 23 SLCs and randomized 1:1 to VR (n=90) or VC (n=96). Primary outcomes – including personal, social, and relational well-being for parents and adult children, and perceived stress and caregiver guilt for adult children – were assessed at baseline, one week post-intervention (primary endpoint), and one and three months post-intervention using intention-to-treat linear mixed models; secondary outcomes captured session engagement via self-report and behavioral coding. Retention was high (89% at post-intervention). Parents with ADRD showed significantly greater improvement in VR than VC for psychological well-being, social connection, and relationship quality, while parents with MCI showed a comparable pattern favoring VC for relationship quality and social connection. Adult children in both conditions reported improved personal, social, and relational well-being, with VR showing more sustained reductions in caregiver guilt through three months. Overall, VR sessions elicited more engagement than VC. These findings indicate that both VR and VC improved well-being and family connection, with VR conferring additional benefits for parents living with dementia and a sustained caregiver-guilt benefit for adult children, suggesting that tailoring technology to cognitive abilities may enhance outcomes in senior care settings.

**Funding:**

National Institute on Aging (NIA), National Institutes of Health (NIH), USA

Individuals living in senior living communities (SLCs) often face barriers to maintaining strong social ties and emotional well-being, particularly when separated from family members by geographic distance. Growing evidence identifies social isolation as a serious health risk for people with cognitive impairment in these settings, contributing to emotional distress, poorer health outcomes, and accelerated cognitive and functional decline.^1–3^ Social isolation has been linked to increased risk of dementia by as much as 50%, as well as higher rates of depression, anxiety, heart disease, and immune dysfunction.^3^ Despite living in a communal setting, 40% of individuals in SLCs report feeling socially disconnected.^4^ Importantly, the quality of connection with family – especially the active presence and involvement of family members – plays a critical role in determining one’s level of vulnerability to social isolation.^5^ With the global population of adults living with Alzheimer’s disease and related dementias (ADRD) projected to reach 87 million by 2031,^6^ there is an urgent need for innovative solutions that can strengthen family connections. Such innovations may not only enhance quality of life for those with cognitive impairments, but also help ease the burden on caregivers and healthcare systems.

Many adult children experience considerable guilt and worry about their parents living in an SLC and managing their care from a distance.^7,8^ There are numerous kinds of SLCs and in the current study this includes people in independent living, assisted living (i.e., those who need help with daily activities but 24-hour care), memory care, and continuing care retirement communities (i.e., a senior living community offering numerous levels of care). Social interventions are needed to help individuals with cognitive and physical impairments thrive in SLCs, while also supporting their ability to maintain meaningful relationships with family members who live far away. In recent years, virtual reality (VR) has emerged as an innovative tool for enhancing quality of life for individuals with cognitive impairments.^9,10^ Multi-user VR, in particular, shows strong potential for strengthening social connections by allowing older adults to engage remotely with family members.^11–15^ The importance of providing individuals in SLCs with access to technologies that facilitate meaningful engagement with family became especially evident during the COVID-19 pandemic, when social distancing heightened social isolation and negatively impacted well-being.^16^

Fully immersive VR has been shown to be safe for older adults with mild to moderate dementia and a promising tool for improving mood, mental health, and quality of life.^11–14^ Although few studies have examined multi-user VR in the context of older adults’ social relationships,^11–14, 17^ early feasibility research suggests that it may reduce feelings of social isolation and strengthen relationships with adult children who participate remotely.^11,12^ Larger clinical trials are necessary, however, to establish the impact of long-distance, multi-user VR on the personal and relational well-being of older adults with cognitive impairments and their family members who use the VR with them from a distance. In addition, it remains unclear how VR compares to other digital modalities, such as video conferencing (VC), which also enable long-distance, social engagement with family. When analyzed alone, several randomized trials have shown that VC can reduce loneliness and improve well-being of socially isolated individuals.^18–20^ During the COVID-19 pandemic, people either in an acute care unit of a hospital, a long-term care community, or skilled nursing facility also reported high satisfaction with VC, especially when technical assistance was provided.^16^ The limited research comparing VR and VC has focused on young adults^21^ or organizational teams.^22,23^ Most of the research involving older adults has explored VR or VC independently, typically in small-scale studies without direct comparison.^24–26^ To our knowledge, the current study represents the first large-scale clinical trial to directly compare VR to VC with older adults or in any context.

The limited research comparing VR to VC in the general population suggests that even though both modalities can effectively support social connection, their respective benefits likely depend on the purpose for which they are used. Studies have shown that the impact of VR, VC, texting, or phone calls on social and psychological outcomes often varies by the task at hand (e.g., creative problem solving, communicating, organizing, task completion, engagement).^22,23^ Some of this work has found no significant differences between VR and VC.^21,22^ However, these studies often lack enriching, immersive, and socially engaging VR content or fail to personalize the experience for individual users.^21^ The differential effects of VR and VC on well-being may depend on multiple factors, including the specific affordances of the modality, the VR platform used, the type of engagement it fosters, and the extent to which it is tailored to characteristics of the target population (e.g., age or degree of cognitive impairment).^20,22,23,27^

Although both VR and VC have the potential to enhance mental health, relational well-being, and overall quality of life for individuals living in SLCs, VR may offer unique advantages or affordances for connecting those with ADRD and their family members who live at a distance. More specifically, VR might offer different levels of cognitive, emotional, social, kinesic, and reminiscence engagement for people living with dementia than VC. Unlike VC, which relies on sustained verbal conversational engagement, which can be challenging for those with memory loss, VR immerses participants in a shared virtual environment, creating a sense of social and physical presence with their loved one.^28–30^ This sensory-rich experience can be especially meaningful for those with cognitive or physical limitations, enabling them to “travel,” participate in novel activities, and engage with the outside world in ways that might otherwise be inaccessible.^11,28^ Moreover, VR has emerged as an important clinical tool for reminiscence therapy in dementia care because of its ability to evoke positive autobiographical memories.^26^ Even though VC can also support reminiscence, VR may foster deeper cognitive, emotional, and physical engagement, potentially amplifying its therapeutic effects.^31^ At the same time, both VR and VC likely enable older adults to maintain relationships with family members who are geographically separated from them, improving well-being and offering valuable tools for care providers. Understanding the specific contexts in which VR or VC is most effective will be essential for maximizing their benefits.

The current clinical trial builds on a prior Phase 1 study testing the efficacy of the Rendever VR platform.^11,30^ Rendever is a fully immersive social VR platform that is currently used in over 900 SLCs across five countries and is designed specifically for older adults, enabling shared virtual experiences and real-time communication between users regardless of physical distance. In an initial feasibility study, we examined whether the platform’s remote features could be used safely and effectively in a sample of 21 parents with mild cognitive impairment (MCI) or mild to moderate ADRD (MMSE ≥ 13) residing in a single SLC, along with a geographically distant family member (primarily adult children).^11,30^ Both parents and their family members found the VR platform to be safe, highly enjoyable, and user friendly.^30^ Observational data revealed that parents were more communicatively and kinesically engaged with their family members during VR sessions compared to a baseline telephone call, especially in sessions with reminiscence-based activities. Interestingly, parents with dementia reported greater immersion in the VR environment than those with MCI, while those with MCI exhibited higher levels of behavioral engagement.^30^ The VR intervention was also associated with significant improvements in the quality of parents’ relationships with their family member, affect, stress, and quality of life, compared to the telephone call.^11^ In addition, family members experienced reductions in negative affect, depressive symptoms, caregiver burden, and improved mental health. Notably, greater social engagement during the VR sessions was associated with better psychological and relational outcomes for both parents and their family members.^11^

Building on this initial work, the current randomized controlled trial – the Technology and Family Thriving (“Thrive”) trial – evaluated the efficacy of a four-week social VR intervention for improving personal and relational well-being in parents with cognitive impairments residing in SLCs and their adult children living at a distance. To isolate the unique effects of immersive VR, we compared it to an active control condition involving VC, a widely available digital tool previously shown to support social connection.^16,20–22^

The overarching goal of the Thrive trial was to examine the impact of a socially enriched VR intervention, compared to a VC control, on well-being and relational outcomes among parents in SLCs and their adult children who lived at a distance. Primary outcomes focused on changes in personal well-being, social well-being, and family relationships for parents and adult children, along with perceived stress and caregiver guilt for adult children. These outcomes were assessed at baseline, one-week post-intervention (the primary endpoint), and at one-month and three-month follow-ups. Secondary outcomes captured real-time emotional, relational, conversational, reminiscence, and kinesic engagement during the remote family sessions. The study also investigated whether the impact of the intervention depended on the parents’ level of cognitive impairment (MCI vs. ADRD). To address these questions, the trial pursued three specific aims.

The first aim (Aim 1) was to evaluate whether the VR intervention would produce greater improvements in the primary outcomes compared to VC. We hypothesized that while both conditions would yield improvements from baseline to post-intervention (due to the shared element of family interaction), parents and adult children in the VR condition would demonstrate significantly greater gains at the immediate post-intervention assessment. Additional follow-up assessments at one- and three-months post-intervention were exploratory, intended to examine the long-term sustainability of these primary benefits.

The second aim (Aim 2) was to evaluate whether the VR condition would produce greater levels of engagement during the weekly family sessions compared to VC. We hypothesized that while both conditions would foster engagement, the VR condition would elicit significantly higher levels of session-based conversational, relational, emotional, and reminiscence engagement (assessed via self-reports and/or behavioral observations) for parents and adult children. We further hypothesized that the VR condition would elicit greater kinesic engagement than VC for parents (assessed via observational and automated coding of session videotapes).

The third aim (Aim 3) was to determine if the parent’s cognitive status (MCI vs. ADRD) moderated these intervention effects on both primary and secondary outcomes. While we had no formal *a priori* hypotheses, given the limited literature, preliminary findings from our Phase 1 feasibility study suggested that VR may offer unique advantages for individuals with more advanced cognitive impairment. Thus, this trial offered a critical opportunity to rigorously evaluate how baseline cognitive status influenced intervention efficacy.

## Methods

### Study design

This study was a stratified, unblinded, parallel-group, multisite, randomized controlled trial in which dyads (parents with MCI or mild to moderate ADRD and an adult child) were randomly assigned to one of two technology-based interventions (virtual reality or video conferencing). Parents were recruited from 23 SLCs in two geographic regions in the United States (Boston, MA, and Santa Barbara, CA, and surrounding areas). To increase the socioeconomic and ethnic diversity of the sample, five low-income senior housing communities were included in the recruitment plan, including one primarily Spanish-speaking site. Residents of participating SLCs were invited to nominate an adult child to participate with them. The trial was conducted between September 2021 and March 2024. Ethical approval was obtained from the Human Subjects Review Committee of the University of California, Santa Barbara. The study was registered on ClinicalTrials.gov (NCT05150990)^1^.

Dyads were randomly assigned to a 4-week intervention consisting of weekly remote family sessions conducted via VR or VC. The 4-week duration was selected to optimize the intervention’s impact based on our pilot data, which demonstrated significant positive outcomes using a shorter, 3-week weekly protocol.^11^ Extending the timeline to 4 weeks was anticipated to provide an even more robust therapeutic experience while maintaining a low participant burden. Video conferencing was chosen as the active control condition because, at the time the trial was initiated, it had become a standard form of communication with family members in SLCs during the pandemic when physical visitation became highly restricted.

### Participant and Public Involvement

While participants were not directly involved in co-designing or conducting the trial protocol, feasibility and user-acceptability feedback from older adults and family members in earlier pilot work informed the session structures and assessment procedures.

### Participant recruitment and eligibility

Recruitment was conducted in collaboration with directors at each participating SLC. Potentially eligible residents were screened in person using a brief interview and the Mini-Mental State Examination, Version 2 (MMSE-2).^32^ Eligible older adults suggested contact information for an adult child who might be willing to participate. Adult children were screened by phone. Dyads were enrolled after both members provided written or digital informed consent. Each participant received US$150 in appreciation for their participation. Additional recruitment details appear in the Appendix (pp.2–3).

Parents were eligible if they were ≥ 50 years old, had MCI (MMSE-2: 25–27) or mild to moderate ADRD (MMSE-2: 13–24),^33^ were fluent in English or Spanish, and had an adult child living ≥ 45 minutes away who was willing to participate. If no adult child was available (5%), another caregiving relative (typically a niece or nephew) could be nominated. Exclusion criteria included MMSE < 13, a history of vertigo, seizures, dementia-related hallucinations, paranoia, aggression, or inability to view images via VR or VC. Adult children were eligible if they were ≥ 18, fluent in English or Spanish, and lived ≥ 45 minutes from the parent’s SLC, with no significant relational conflict or history of abuse. They were excluded for vertigo, seizures, or visual impairments that could interfere with participation.

### Randomization and masking

Dyads were randomized 1:1 to the VR or VC group using stratified block randomization, with strata defined by parent’s cognitive status (MCI vs. ADRD) and region (Boston vs. California) to ensure balanced allocation. The random allocation sequence was generated prior to study initiation (via the *randomizeR* package in R) by a PI who was not involved in direct participant recruitment or enrollment. Due to the nature of the intervention, participants and research staff were not masked to group assignments.

### Procedures

**Figure 1.**
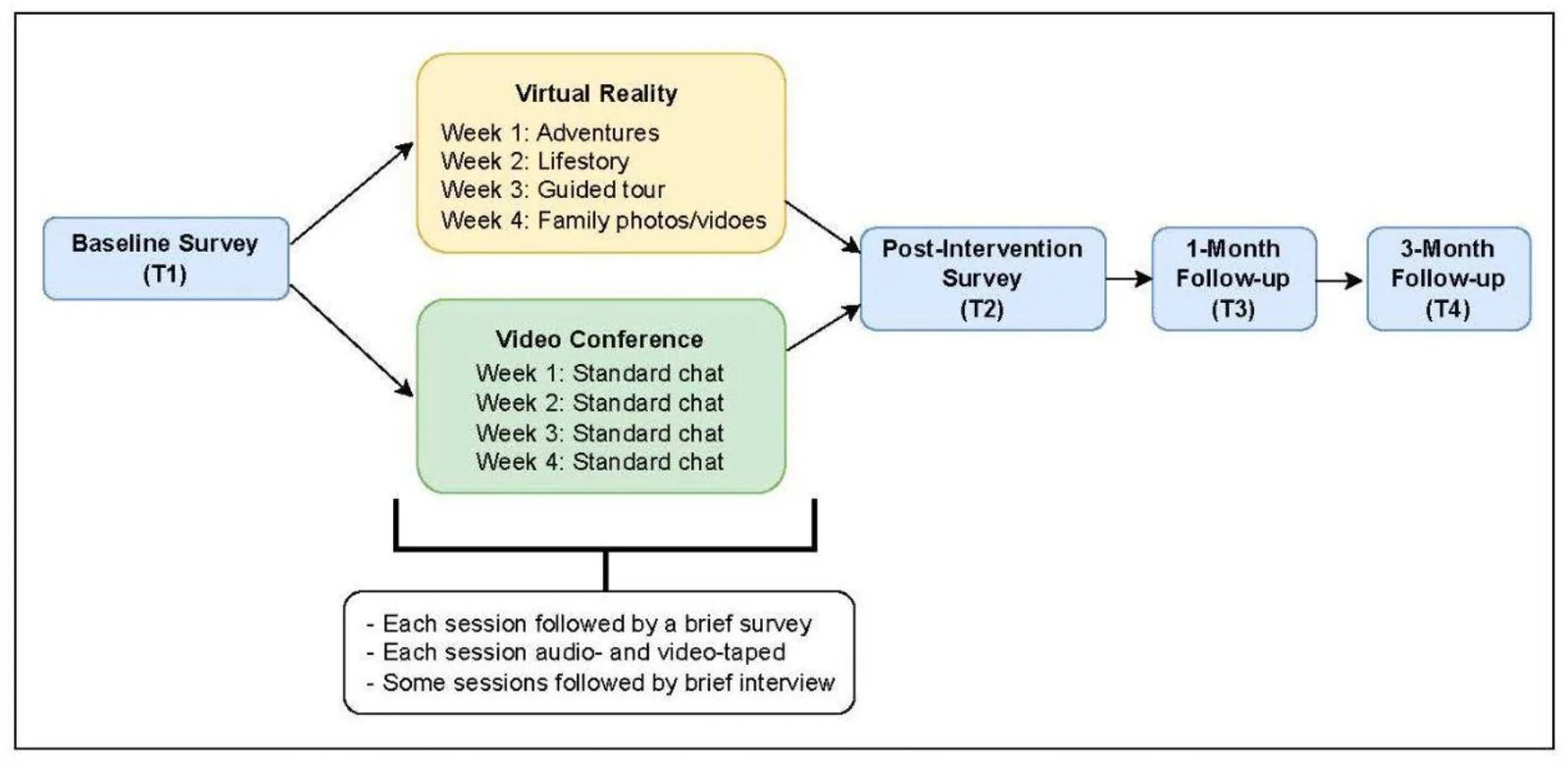
Study design and timeline

The study timeline is shown in Figure 1. Dyads completed a baseline survey, followed by four weekly VR or VC sessions. Brief secondary outcome surveys were completed after each weekly technology session. Primary outcomes were assessed within one week of the final session, and again at one- and three-month follow-ups. Older adults completed paper surveys in person, face-to-face with a member of the research team; adult children completed surveys online. With permission, sessions were audio-recorded and parents were video-recorded. These recordings were later coded for various forms of engagement as secondary outcomes.

Parents participated in weekly VR/VC sessions from a private space within their SLC (or their room), with support from the research team. Adult children joined from home and were trained remotely to use the platforms. All adult children received a welcome packet; those in the VR group were also mailed a VR headset. Sessions lasted approximately 20 minutes. Most participants (95% of parents, 90% of adult children) consented to recording, resulting in 372 hours of audio/video data. Below is a brief description of each intervention condition (see the Appendix for details, pp.6–8).

Dyads assigned to the VR condition used Rendever’s social engagement platform, designed specifically for older adults. A researcher helped place the headset (Pico G2 4KE) on the parent and navigated the VR experience via a tablet. Adult children participated remotely, with a researcher available by phone for support. VR headsets included a built-in microphone that allowed for shared experiences and real-time communication between parents and adult children. In Session 1 (*Virtual Adventures*), dyads selected from more than 80 travel adventures (e.g., safari, hot air balloon ride). In Session 2 (*Virtual Life Story*), researchers guided dyads through eight meaningful locations from the older adult’s past (e.g., childhood homes, family vacation spots), with addresses uploaded in advance by the adult child via Rendever’s secure family portal. Session 3 (*Virtual Guided Tour*) offered a researcher-led exploration of a global destination selected by the dyad (e.g., hiking Machu Picchu, touring Rome). In Session 4 (*Family Photos and Videos*), adult children uploaded 15+ photos and 1–2 videos to the family portal, which the dyad viewed together in a virtual family room, seated side-by-side on a couch through their personalized avatars.

Dyads assigned to the VC condition followed the same weekly schedule and completed the same surveys as those in VR, but engaged in unstructured conversations via Zoom. No guided activities were provided; participants were simply encouraged to talk as they normally would. For parents, the research team set up and operated the Zoom sessions using a laptop; adult children received a link via email. Once the session began, the researcher stepped away to allow privacy but remained nearby to offer support if needed.

Data on adverse events and potential harms were collected non-systematically through spontaneous participant self-reports and staff observation during scheduled study contacts. All adverse events were reviewed by Principal Investigators and an independent safety monitor to assess severity and potential relatedness to the intervention.

### Outcomes

Demographic and health characteristics were collected at intake. Primary outcomes included changes in self-reported personal and social well-being and family relationships for parents and adult children, along with caregiver guilt and perceived stress in adult children. These outcomes were assessed at baseline, one-week post-intervention (primary endpoint), and at one-month and three-month follow-ups. Secondary outcomes focused on relational, emotional, conversational, reminiscence, and kinesic engagement during remote family sessions. Brief descriptions of each measure are provided below; full details (items, response formats, and reliabilities) are presented in the Appendix (pp.8–18). Correlations among the outcomes and covariates are provided in the Appendix (Tables S1–4, pp.21–25).

To reduce redundancy and the number of hypothesis tests, we created composite indices for some of the primary outcomes by standardizing and averaging conceptually related and highly correlated measures. Specifically, we grouped outcomes in three domains: *psychological well-being* (quality of life for parents, thriving, positive affect), *psychological distress* (depression, negative mental health symptoms, negative affect), and *relationship quality* (closeness, satisfaction, communal coping). This approach enhances statistical efficiency, reduces measurement error and Type I error risk, and provides a more reliable and holistic assessment, particularly valuable when working with individuals with cognitive impairments. Although we also had several indicators of social well-being for the parents (loneliness, social network support, social connection), these were not highly intercorrelated and were therefore not combined into a single index (see Table S1).

#### Primary outcomes - Parents

(a) ***Psychological well-being*** was assessed by standardizing and averaging three measures: quality of life, thriving, and positive affectivity. Quality of life was measured with the 13-item Quality of Life in Alzheimer’s Disease (QoL-AD) scale.^34^ Thriving was measured with nine items from the Brief Inventory of Thriving and two items (one adapted) from the Comprehensive Inventory of Thriving to assess learning something new and autonomy, which are especially relevant for this study and not captured in the brief measure.^35^ Positive affectivity was assessed with six emotion terms from the Positive and Negative Affect Schedule (PANAS),^36^ measuring positive emotions experienced over the past week. Together, these measures reflect overall life satisfaction, purpose, and emotional vitality. (b) ***Psychological distress*** was assessed by standardizing and averaging three measures: depressive symptoms, mental health symptoms, and negative affectivity. Depressive symptoms were measured using the 15-item Geriatric Depression Scale Short Form (GDS-SF).^37^ Mental health symptoms were assessed with the 5-item Mental Health Inventory (MHI-5),^38^ a brief screening tool for mood and anxiety disorders. Negative affectivity was measured using six emotion terms from the PANAS, capturing unpleasurable emotional states during the past week. Together, these measures reflect general psychological distress and negative mood. (c) ***Loneliness*** was assessed with four items adapted from the UCLA Loneliness Scale^39^ and Hughes et al.’s short scale for measuring loneliness.^40^ (d) ***Social network support*** was assessed with the 6-item Lubben Social Network Scale (LSNS-6),^41^ which captures the size and closeness of family and friend networks. (e) ***Social connection*** was measured with seven items developed for this study to assess interpersonal engagement and connection to the broader community and world at large (e.g., satisfaction with social network, sense of oneness with the world, enjoyment of group participation and new friendships). (f) ***Relationship quality with their child*** was assessed by standardizing and averaging three measures: relationship closeness, relationship satisfaction, and communal coping. Closeness was assessed with four items from the Unidimensional Relationship Closeness Scale.^42^ Satisfaction was measured using three items adapted from Huston et al.’s satisfaction scale^43^ and the Couples Satisfaction Index (CSI-4).^44^ Communal coping, which reflects shared efforts in managing stress, was assessed with three items from the Communal Coping Scale.^45^ Together, these measures capture emotional connection, satisfaction, and shared coping in the face of life’s challenges.

#### Primary outcomes - Adult children

(a) ***Psychological well-being*** was assessed by standardizing and averaging two scales, thriving and positive affectivity (PANAS), described above. (b) ***Psychological distress*** was assessed by standardizing and averaging three scales, depression, mental health symptoms, and negative affectivity. Depression was measured with the 10-item Center for Epidemiological Studies Depression Scale Revised Short Form (CESD-R-10).^46^ Mental health symptoms were measured with the MHI-5, and negative affectivity with the PANAS, described above. (c) ***Perceived stress*** was measured with Cohen et al.’s 4-item Perceived Stress Scale,^47^ focusing on experiences of feeling overwhelmed and lacking control. (d) ***Caregiver guilt*** was measured with two items from Wells et al.’s Caregiver Guilt Scale^48^ measuring guilt and worry about having a parent in a SLC, supplemented by three items written for this study to measure guilt related to lack of engagement due to geographic separation (e.g., “I feel bad that I canť visit my parent more often”). (e) ***Social connection*** was measured with five items developed for this study to assess feelings of social connection to the community and broader world, similar to the older adult version. (f) ***Relationship quality with their parent*** was assessed by standardizing and averaging the same three scales (closeness, satisfaction, and communal coping) used for parents, but reworded to reflect the adult child’s perspective.

#### Secondary outcomes

Secondary outcomes focused on the participants’ engagement during the weekly VR/VC sessions and included self-report measures as well as variables coded from audio and video-recordings. All secondary outcomes were aggregated across the four sessions. For measures that included multiple items, items were averaged together within each session to form a session composite. Then, the four session composites were averaged together to form an overall index of engagement across the intervention period. Brief descriptions are provided below; full details (items, response formats, reliabilities) are available in the Appendix (p.14–18).

##### Self-report measures.

In post-session surveys, participants provided their immediate responses to the weekly VR and VC sessions. These self-report measures were designed specifically for this study to capture emotional, relational, and reminiscence experiences during each session. Adapted from our Phase 1 trial,^11,30^ measures included: (a) *positive and negative emotion* (using the same PANAS items from the primary outcomes, but asking how they felt during the session), (b) *relational connection*, (c) *relational strain*, (d) enjoying *learning something new* together, (e) enjoying *reminiscence* together, and (f) *sharing special times*. These subscales drew from Burgoon and Hale’s Relational Communication Topoi Scale^42^ and study-specific items (Appendix, pp.14–15).

##### Observational coding.

Engagement during VR and VC sessions was coded from recorded verbal and nonverbal behavior (Appendix, pp.16–17). Thirteen trained research assistants who were blind to the study hypotheses used adapted versions of two established coding schemes to rate four types of engagement: conversational, reminiscence, emotional, and kinesic.

The first set of ratings was completed separately for parents and adult children based on audio recordings. Adapted from Burgoon and Hale’s coding system,^49^ these items measured conversational dynamics, including (a) *conversational engagement* (e.g., “the older adult/adult child was involved in the conversation”) and (b) *conversational lead* (e.g., “the older adult/adult child did more talking than listening”). Additionally, (c) a custom item developed for this study measured *reminiscence,* capturing moments when participants reflected on positive past experiences (“the parent/adult child reminisced positive moments from the past”).

The second set of ratings was based on video recordings of parents only (adult children were not video-recorded). Adapted from Guerrero’s nonverbal coding system,^50^ these items measured parent-specific emotional and kinesic engagement: (d) *emotional warmth* (e.g., smiling, being friendly, sounding content, pleasant, warm), (e) *emotional vitality* (e.g., laughing, sounding happy, being animated, full of life), and (f) *kinesic engagement* (e.g., gestures, limb and trunk movement).

##### Computer coding.

To supplement the human observational coding, a second, objective measure of kinesic engagement was obtained via automated software tracking. Using methods validated in our Phase 1 pilot,^11,30^ video recordings of parents’ VR and VC sessions were coded for kinesic engagement using *OpenPose*, an open-source computer vision software program that converts raw visual data into structural representations of human movement.^51^ The tracking algorithm identified a subset of 10 primary anatomical keypoints across the participant’s face, body, hands, and feet to calculate the frequency and magnitude of their physical movement during the sessions, with higher scores reflecting more active and sustained motion. Additional information regarding this analysis appears in the Appendix (p.18).

### Sample size calculation

An a priori power analysis using simulation methods for multilevel regression was conducted. Effect sizes were estimated from the Phase I pilot study.^11,30^ Attrition was estimated at 15%. A target sample size of 192 dyads (96 per group) was calculated to detect a minimal expected effect size (Cohen’s *d*=0.20), corresponding to a between-group difference in within-person change scores of 0.2 standard deviations, with 85% power and a two-sided alpha of 0.05. Recruitment was delayed due to public health concerns during the later stages of the COVID-19 pandemic, which temporarily limited access to several participating SLCs. To complete the trial within the funding window, enrollment was closed after 186 dyads.

### Statistical analysis

All analyses were conducted in IBM SPSS Statistics, Version 30; figures were created in R. Because different outcomes were assessed for parents and adult children, models were estimated separately for each group. All models for parents controlled for age, sex, number of chronic health conditions; all models for adult children controlled for age and sex. All statistical tests were two-sided, and a *p*-value of less than .05 was considered statistically significant. Given the modest sample size and the use of planned, hypothesis-driven model testing, no corrections for multiple comparisons were applied, in order to preserve statistical power.

#### Primary outcome analyses (Aims 1 and 3)

*Primary outcomes* were analyzed using a series of intention-to-treat linear mixed models (LMMs) with restricted maximum likelihood estimation (REML). All LMMs included a random intercept for participant to account for repeated observations over time. Missing outcome data were handled directly within the LMM framework under the Missing at Random (MAR) assumption, allowing REML estimation to utilize all available data from all enrolled participants without requiring listwise deletion or data imputation.

To address Aims 1 and 3, we estimated two sets of LMMs. The first set of LMMs examined changes in well-being and family relationships from baseline (T1) to post-intervention (T2), while also examining whether these effects were moderated by the parent’s cognitive status. Fixed effects included Time, Technology Condition (VR vs. VC), Cognitive Status (MCI vs. ADRD), and their two-way and three-way interactions. The key effect was the Time × Technology Condition interaction, where a significant interaction effect (*p*<.05) would indicate that within-person changes over time differed between intervention groups. To evaluate moderation by cognitive status, we examined the three-way Time × Technology Condition × Cognitive Status interaction, where a significant interaction effect (*p*<.05) would indicate that intervention-related changes over-time varied by cognitive status. The main effect of Time and the Time × Cognitive Status interaction were also examined to assess overall pre- to post-intervention change across conditions.

Guided by the study aims and hypotheses, we conducted pairwise contrasts using estimated marginal means to calculate within-group change scores (T1 vs. T2) for each technology condition (VR and VC) and cognitive status group (MCI and ADRD), as appropriate. Cohen’s *d* effect sizes were calculated by dividing the difference-in-differences (hereafter DiD) contrast by the standard deviation of the outcome at baseline. Confidence intervals for these effects were derived from the standard errors of the model-based contrasts.

The second set of LMMs examined whether intervention effects on primary outcomes were sustained beyond the intervention period at the 1-month (T3) and 3-month (T4) follow-ups. These models paralleled the first set but included all four time points (T1 to T4) and incorporated a correction for autocorrelation among time points. Analyses focused on changes from baseline to T3 and T4. Pairwise contrasts compared each post-baseline assessment with baseline (T1 vs. T2, T1 vs. T3, and T1 vs. T4) within each technology condition and cognitive status group, as appropriate.

#### Secondary outcome analyses (Aims 2 and 3)

To test the effects of the intervention on *secondary outcomes* – specifically, parent and child engagement averaged across the remote family sessions – we conducted a series of two-way between-subjects ANCOVAs (Technology Condition × Cognitive Status).

## Results

### Recruitment and retention

**Figure 2.**
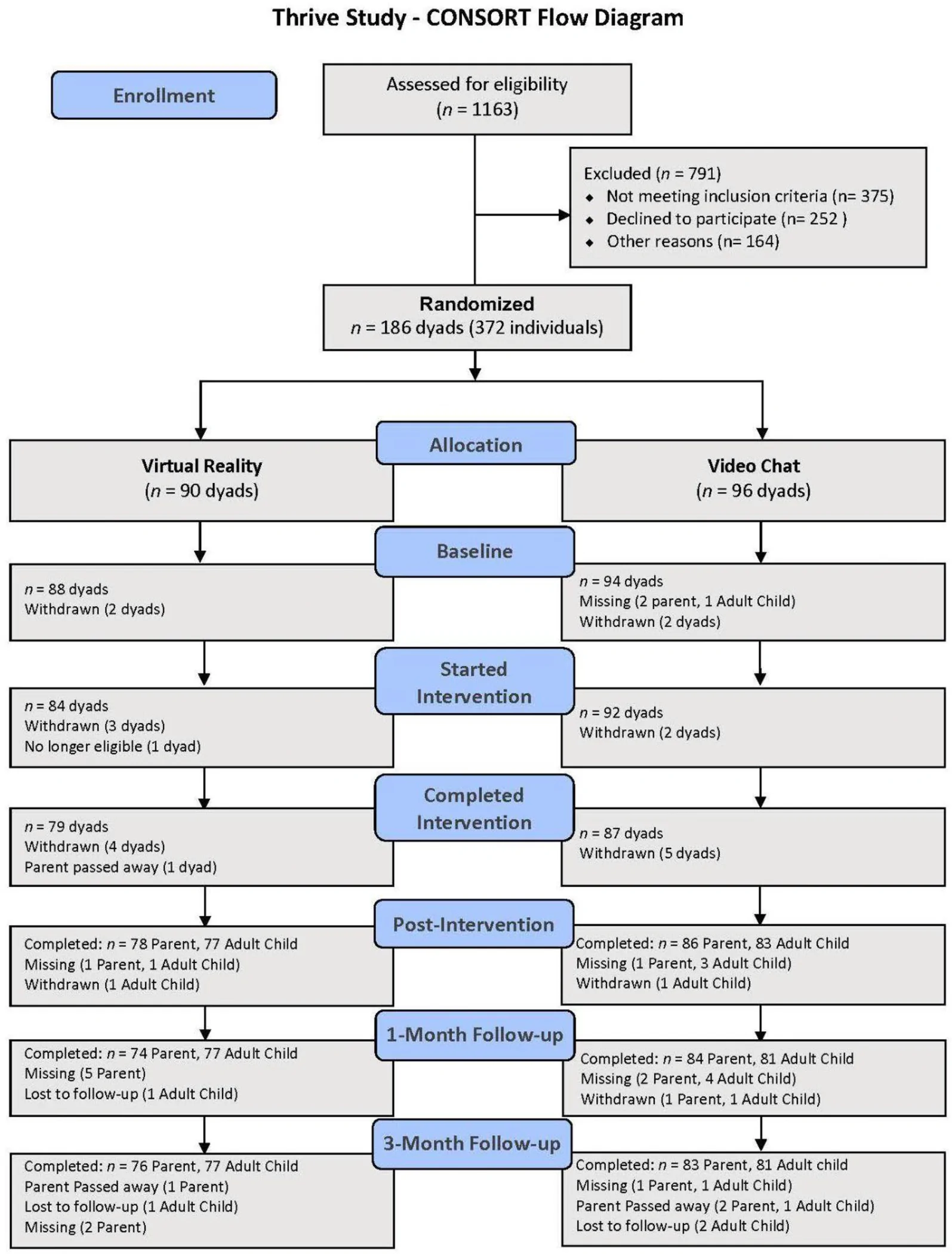
CONSORT diagram of participant flow

Recruitment occurred between September 1, 2021 and December 1, 2023. Of the 1,163 parents and adult children approached (Figure 2), 791 were excluded (375 not interested, 252 ineligible, 164 other). A total of 186 dyads (372 individuals) enrolled and were randomized to VR (*n*=90 dyads) or VC (*n*=96 dyads). Intervention adherence was high, with 166 dyads (89%) successfully completing the remote VR/VC family sessions. Following the intervention, individuals remained eligible for subsequent assessments independently, regardless of their dyadic partner’s survey completion. At post-intervention, survey retention was 89% for parents (*n*=166) and 88% (*n*=164) for adult children. At the 3-month follow-up, survey retention remained high at 88% for parents (*n*=164) and 87% for adult children (*n*=161). These retention rates successfully met our a priori power analysis target, which anticipated an 85% retention rate resulting in at least 164 completed dyads.

Attrition did not differ by intervention group. Attrition and missing data are summarized in Figure 2 and detailed in the Appendix (pp.3–5). Briefly, 20 dyads and 12 individuals withdrew or were lost to follow-up: 10 dyads withdrew before the intervention began (mostly due to time or health), 10 during the intervention (mostly due to health; two to family tensions; one to physical discomfort using VR; one to parental death). Eight individuals withdrew post-intervention (primarily due to survey burden or parental illness/death); four were lost to follow-up. All health-related events were unrelated to the intervention. Participants who withdrew after baseline were included in intention-to-treat analyses. Participants who missed one or more surveys but remained in the study were included in all relevant analyses.

There were no serious or unexpected adverse events or harms. However, three dyads in the VR condition withdrew due to discomfort: one parent experienced physical discomfort using VR and two families experienced relational strain due to prior family history.

### Baseline characteristics

Baseline characteristics of the sample are presented in Table 1. Parents ranged in age from 60 to 99 (*M* = 85.8, *SD* = 7.5) and were mostly female (69%), widowed (64%), and of White ethnicity (96%). MMSE scores ranged from 13 to 17 (*M*=23.0, *SD*=3.72); approximately half had MCI (46%, *N*=85) and half had mild to moderate ADRD (54%, *N*=101). Overall, they were highly educated, with nearly two-thirds (62.3%) holding a bachelor’s or graduate/advanced degree. A smaller portion had completed some college or vocational training (23.7%) or reported a high school education or less (10.2%, with 3.8% unreported). Two parents completed surveys in Spanish; all others completed them in English.

Adult children (or other family members, 5%) were, on average, 56.4 years old (*SD*=10.2), mostly female (60%) and of White ethnicity (95%). They lived in 29 states in the U.S. and 8 countries. Overall, they were very highly educated, with the vast majority (83.8%) holding a bachelor’s or graduate/advanced degree. Only 10.8% completed some college or vocational training, and only 4.8% reported a high school education or less. All adult children completed surveys in English.

Baseline levels on the outcome variables showed moderate psychological well-being (quality of life for parents, thriving, positive affectivity); relatively mild psychological distress (depression, mental health symptoms, negative affectivity); relatively low loneliness and moderate social network support and social connection for parents; relatively low perceived stress and caregiver guilt for adult children; and relatively high relationship quality (closeness, satisfaction, communal coping).

### Primary outcomes for parents

#### Baseline to post-intervention

Table 2 provides the fixed-effects test statistics for the first set of LMMs examining change from baseline (T1) to post-intervention (T2) among parents. Table 3 presents covariate-adjusted mean change (95% CIs), difference-in-differences (DiD) contrasts, and corresponding effect sizes for primary outcomes T1 to T2 by Technology Condition and Cognitive Status. Estimated mean changes in the overall sample appear in the Appendix Table S5 (pp.26–27). Extended Data Figure 1 illustrates key findings for each outcome variable.

There were no significant Time × Technology Condition interactions for any primary outcome, indicating no evidence that the VR and VC conditions differed in average change over time when collapsing across Cognitive Status (Aim 1). However, significant Time × Technology Condition × Cognitive Status interactions were observed for psychological well-being, social connection, and relationship quality. These interactions indicate that differences between VR and VC in change over time varied according to parents’ Cognitive Status (Aim 3).

##### Psychological well-being.

A significant Time × Technology Condition × Cognitive Status interaction was observed for psychological well-being (*p*=.014). Among parents with ADRD, psychological well-being improved significantly in the VR condition (*M*_diff_=.34, *p*<.001) but not in the VC condition (*M*_diff_=.05, *p*=.545). The DiD contrast was significant (*M*_DiD_=.29, *p*=.013), indicating greater improvement in VR than in VC. In contrast, among parents with MCI, psychological well-being did not change significantly in either the VR condition (*M*_diff_=−.09, *p*=.275) or the VC condition (*M*_diff_=.03, *p*=.728), and the DiD contrast was not significant (*M*_DiD_=−.12, *p*=.304). The difference between the ADRD and MCI DiD contrasts (DiD_ADRD_ – DiD_MCI_= .29–[−.12]=.41, *p*=.014) explains the three-way interaction.

##### Psychological distress.

Results showed only a main effect of Time, with psychological distress decreasing from baseline to post-intervention across technology conditions and cognitive-status groups (*M*_diff_=−.10, *p*=.027; Table S5).

##### Loneliness.

No significant main effects or interactions were observed for loneliness.

##### Social network support.

A significant Time × Cognitive Status interaction was observed for social network support (*p*=.022). When collapsing across technology conditions, parents with ADRD showed a significant increase in social network support from baseline to post-intervention (*M*_diff_=.19, *p*=.026), whereas parents with MCI showed no significant change over time (*M*_diff_=−.10, *p*=.296; Table S5).

##### Social connection.

A significant Time × Technology Condition × Cognitive Status interaction was observed for social connection (*p*=.034). Follow-up analyses showed a crossover pattern in the relative effects of VR and VC by Cognitive Status. Among parents with ADRD, social connection improved significantly in the VR condition (*M*_diff_=8.82, *p*=.018) but not in the VC condition (*M*_diff_=−1.96, *p*=.585). The DiD contrast was significant (*M*_DiD_=10.77, *p*=.037), indicating greater improvement in VR than in VC. In contrast, among parents with MCI, social connection did not change significantly in the VR condition (*M*_diff_=2.71, *p*=.465) but improved significantly in the VC condition (*M*_diff_=7.46, *p*=.037). However, the DiD contrast was not significant (*M*_DiD_=−4.76, *p*=.355), indicating insufficient evidence that change differed between the two technology conditions among parents with MCI. The difference between the ADRD and MCI DiD contrasts (DiD_ADRD_–DiD_MCI_=10.77–[−4.76]=15.53, *p*=.034) explains the significant three-way interaction.

##### Relationship quality.

A significant Time × Technology Condition × Cognitive Status interaction was observed for relationship quality (*p*=.046). Follow-up analyses showed a crossover pattern in the relative effects of VR and VC by Cognitive Status. Among parents with ADRD, relationship quality improved significantly in the VR condition (*M*_diff_=.26, *p*=.004) but not in the VC condition (*M*_diff_=.07, *p*=.402). However, the DiD contrast did not reach significance (*M*_DiD_=.19, *p*=.124). Among parents with MCI, the pattern reversed: relationship quality did not change significantly in the VR condition (*M*_diff_=.04, *p*=.668) but improved significantly in the VC condition (*M*_diff_=.20, *p*=.022). The corresponding DiD contrast was also not significant (*M*_DiD_=−.16, *p*=.196). Although neither Cognitive Status group showed a significant DiD contrast – reflecting smaller within-group effect sizes relative to the total crossover spread evaluated by the omnibus test – the difference between the ADRD and MCI DiD contrasts (DiD_ADRD_–DiD_MCI_=.19−[−.16]=.35, *p*=.046) explains the significant three-way interaction.

#### Longitudinal follow-ups

The second set of LMMs examined whether changes observed immediately after the intervention were maintained at the 1- and 3-month follow-ups for parents. Extended Data Table 1 presents the fixed-effects test statistics from the LMMs spanning T1 through T4. Extended Data Table 2 presents covariate-adjusted mean changes with 95% CIs, DiD contrasts, and corresponding effect sizes for primary outcomes from baseline (T1) to each post-baseline assessment (T2–T4) by Technology Condition and Cognitive Status. Estimated mean changes in the overall sample are presented in Appendix Table S6 (pp.28–30). Extended Data Figure 2 illustrates key findings for each outcome over time. Overall, many of the beneficial changes observed at T2 were partially maintained during follow-up (Aims 1 and 3).

##### Psychological well-being.

The three-way interaction of Time × Technology Condition × Cognitive Status remained significant in the T1–T4 model (*p*=.044). As reported above, parents with ADRD showed significantly greater improvement in VR than in VC at post-intervention. This between-group comparison remained significant at the 1-month follow-up (*M*_DiD_=.28, *p*=.040), but was no longer significant at 3-months (*M*_DiD_=.13, *p*=.347). Among parents with MCI, no significant within-condition changes or between-condition DiD contrasts were observed at either follow-up.

##### Psychological distress.

The main effect of Time remained significant in the T1–T4 model (*p*=.044). However, the reduction from baseline observed in the overall sample at post-intervention was not evident at either the 1-month follow-up (*M*_diff_=−.05, *p*=.380) or 3-month follow-up (M_diff_=.04, *p*=.468; Table S6). Thus, there was no evidence that the initial reduction in psychological distress was maintained during follow-up. No significant Technology Condition or Cognitive Status effects were observed at either follow-up.

##### Loneliness.

Consistent with the absence of significant effects at post-intervention, no significant within-condition or between-condition DiD contrasts were observed at the 1- or 3-month follow-ups.

##### Social network support.

The Time × Cognitive Status interaction remained significant in the T1–T4 model (*p*=.017). Among parents with ADRD, the improvement from baseline observed at T2 was not evident at the 1-month follow-up (*M*_diff_=.02, *p*=.859), but was again significant at the 3-month follow-up (*M*_diff_=.19, *p*=.039; Table S6). These estimates were calculated while collapsing across technology conditions. No significant changes were observed among parents with MCI at any assessment.

##### Social connection.

The Time × Technology Condition × Cognitive Status interaction was not significant in the T1–T4 model (*p*=.244). Given the prespecified focus on Cognitive Status as a moderator, simple-effects analyses were nevertheless explored within each Cognitive Status group and should be interpreted with caution. Among parents with ADRD, the VR advantage observed at post-intervention was no longer significant at the 1-month (*M*_DiD_=8.09, *p*=.145) or 3-month follow-up (*M*_DiD_=6.29, *p*=.274). However, parents with ADRD in the VR condition continued to show significant within-group improvement from baseline at the 3-month follow-up (*M*_diff_=9.27, *p*=.027). No significant DiD contrasts were observed among parents with MCI at either follow-up.

##### Relationship quality with child.

The Time × Technology Condition × Cognitive Status interaction remained significant in the T1–T4 model (*p*=.017). The cross-over pattern observed at post-intervention persisted at the 1-month follow-up: parents with ADRD showed greater improvement in VR than in VC (*M*_DiD_=.28, *p*=.026), whereas parents with MCI showed greater improvement in VC than in VR (*M*_DiD_=−.28, *p*=.027). At the 3-month follow-up, the DiD contrast was not significant within either Cognitive Status group.

### Primary outcomes for adult children

#### Baseline to post-intervention

Table 4 presents the fixed-effects test statistics for the first set of LMMs (T1 to T2) for adult children. Table 5 provides the covariate-adjusted mean differences from baseline to post-intervention (T1 to T2; 95% CIs) for all primary outcomes. Results are reported for the overall sample and stratified by technology condition. Extended Data Figure 3 illustrates key findings for each outcome variable.

There were no significant Time × Technology Condition interactions (Aim 1), nor were there any significant two-way or three-way interactions involving parent’s cognitive status (Aim 3). Thus, unlike the results for parents, the immediate benefits of the intervention for adult children did not differ based on the type of technology used or their parent’s level of cognitive impairment.

However, main effects of Time emerged across all outcomes. Regardless of whether they used VR or VC, adult children reported significant improvements from baseline in psychological well-being (*M*_diff_=.12, *p*=.010), social connection (*M*_diff_=4.64, *p*=.017), and relationship quality (*M*_diff_=.13, *p*<.001), along with reductions in psychological distress (*M*_diff_=−.13, *p*=.018), perceived stress (*M*_diff_=−.14, *p*=.005), and caregiver guilt (*M*_diff_=−.08, *p*=.046).

#### Longitudinal follow-ups

The second set of LMMs examined whether the within-person improvements observed across the full sample at post-intervention were sustained at the 1- and 3-month follow-ups for adult children. Extended Data Table 3 presents the fixed effects test statistics. Extended Data Table 4 presents covariate-adjusted mean differences comparing baseline (T1) to all post-intervention assessments (T2 to T4; 95% CIs) for all primary outcomes, reported for the overall sample and stratified by technology condition. Extended Data Figure 4 illustrates key findings for each outcome over time.

The main effect of Time remained significant across all six outcomes, with benefits sustained across most domains through both follow-up periods. Improvements from baseline in psychological well-being and relationship quality observed at post-intervention were maintained at both the 1-month (*p*=.018, *p*=.002, respectively) and 3-month (*p*=.031, *p*=.006, respectively) follow-ups. Reductions in perceived stress and caregiver guilt were also maintained across both follow-ups (all *p*s<.01). Increases in social connection were maintained at 1-month (*p*=.002) but not at 3-months (*p*=.371). Similarly, reductions in psychological distress were sustained at 1-month (*p*=.002) but only marginally significant at 3-months (*p*=.069).

Consistent with the immediate post-intervention findings, no significant Technology Condition or Cognitive Status effects emerged at the 1- and 3-month follow-ups for adult children (Aims 1 and 3), with one exception: a Time × Technology Condition interaction for caregiver guilt (*p*=.034). Adult children in the VR condition showed significant reductions in guilt at both the 1- and 3-month follow-ups (*p*<.001 for both), while those in the VC condition showed no significant change at either timepoint (Extended Data Table 4). However, the pairwise DiD contrasts comparing conditions directly did not reach statistical significance at 1-month (*M*_DiD_=−.12, *p*=.159), but was marginally significant at 3-months (*M*_DiD_=−.14, *p*=.096), reflecting smaller effect magnitudes at individual timepoints relative to the cumulative trajectory evaluated by the omnibus interaction test.

### Secondary outcomes for parents and adult children: Engagement during remote family visits

Table 6 presents mean differences and corresponding effect sizes for self-reported as well as observational- and computer-coded engagement during remote family visits across the VR and VC conditions for both parents and adult children. Full statistical outputs are provided in Extended Data Table 5. In general, results revealed greater overall engagement for participants in the VR condition compared to the VC condition (Aim 2). None of these effects were significantly moderated by the parent’s Cognitive Status (Aim 3); however, engagement differed by Cognitive Status across several outcomes (see Extended Data Table 5).

#### Self-reported engagement during remote family sessions

Among parents, there were no significant condition differences in positive emotion or relational connection. However, compared to those in the VC condition, parents in the VR condition were significantly more likely to report having learned something new (*p*<.001), enjoying reminiscing about old times (*p*<.001), and enjoying sharing special moments with their child (*p*=.003). Parents in VR also reported significantly less relationship strain during their interactions (*p*=.006) and marginally lower levels of negative emotion (*p*=.061) than those in VC.

Adult children also reported more favorable experiences in VR relative to VC. Compared to the VC condition, adult children using VR reported significantly greater positive emotion (*p*=.009) and were significantly more likely to report that they learned something new (*p*<.001), enjoyed reminiscing about old times (*p*=.002), and enjoyed sharing special moments with their parent (*p*=.013). No significant group differences emerged for adult children’s negative emotion, relational connection, or relational strain.

#### Observational and computer coding of engagement during remote family sessions

Audio coding of parent and child communication behaviors indicated no condition differences in overall conversational engagement. However, both parents and adult children engaged in significantly higher levels of reminiscence in the VR condition compared to the VC condition (*p*<.001 for both). Additionally, conversational dynamics varied by technology: parents were significantly more likely to take the conversational lead during VR sessions (*p*=.034), whereas adult children more often took the lead during VC sessions (*p*=.002).

Observational coding of parents revealed no significant condition differences in emotional warmth (e.g., smiling, being friendly, sounding content). However, parents in the VR condition exhibited significantly greater expressions of emotional vitality (e.g., laughing, being excited, full of life, conveying wonder) compared to those in the VC condition (*p*=.023). In addition, both human observational coding (*p*=.001) and automated computer coding (*p*=.009) showed that parents exhibited significantly more kinesic engagement – characterized by active movement of the head, body, and limbs – during VR sessions relative to VC sessions.

## Discussion

To our knowledge, this is the first clinical trial to compare the impact of remote, multi-user VR and VC on personal and relational well-being for parents living with cognitive impairments and their adult children. Considered together across our three aims, four conclusions emerge. First, VR and VC produced meaningful improvements in psychological well-being, family relationship quality, and social connection for parents and adult children alike, indicating that structured, technology-facilitated family contact is beneficial for both generations regardless of platform (Aim 1). Second, for several outcomes, the relative benefit of VR versus VC for parents differed by cognitive status, with VR showing clearer advantages for parents with mild to moderate ADRD and VC showing clearer advantages for parents with MCI. Some of these effects were sustained at the one- and three-month follow-ups (Aims 1 and 3). Third, this moderation by cognitive status was largely specific to parents; adult children benefited similarly regardless of parent’s cognitive status and which technology was used, with one notable exception – reductions in caregiver guilt were sustained over time only in the VR condition, through the three-month follow-up (Aim 1). Fourth, VR and VC fostered different forms of engagement. VR elicited more parent-led conversation, whereas VC elicited more child-led conversation. VR also promoted more positive shared experiences with a family member, more positive emotions for adult children, as well as more emotional vitality, greater kinesic engagement, and less relational strain for parents (Aim 2). We discuss each of these findings in turn below, along with their implications for family-centered technology interventions in SLCs.

Among parents, intervention effects differed by cognitive status for a subset of outcomes, although the strength of evidence varied across outcomes. The clearest evidence emerged for psychological well-being, with parents with ADRD showing significant improvement in VR but not VC and a statistically significant difference in change between conditions. Social connection showed a similar pattern among parents with ADRD, who improved significantly more in VR than in VC. Although parents with MCI in the VC condition showed a significant increase in social connection, this improvement did not significantly exceed the change observed in VR. For relationship quality with their adult child, parents with ADRD in VR and parents with MCI in VC showed significant improvement from baseline, and the overall three-way interaction was significant. However, the direct VR-versus-VC contrast was not statistically significant within either cognitive-status group. We therefore interpret this crossover pattern as suggestive rather than conclusive. Separately, parents with ADRD showed a significant increase in social network support from baseline to post-intervention regardless of technology condition.

The current findings replicate and extend our prior feasibility study, which suggested that immersive social VR with a family member (compared to a baseline telephone call) may be especially valuable for improving psychological and relational well-being for older adults with mild to moderate ADRD.^11,30^ In that pilot work, older adults living with dementia reported greater immersion in the VR with their family member than older adults with MCI.^30^ The immersive nature of multi-user VR, combined with its capacity to evoke autobiographical memories with a loved one with similar shared experiences, may make it particularly effective for people living with dementia. One possible explanation for why parents with MCI showed some advantages in VC over VR is that their relatively stronger cognitive and communicative abilities allowed VC’s conversation-based format to support richer, smoother exchanges with their adult children.

For parents, several of the benefits observed immediately post-intervention persisted beyond the four-week intervention window. Among parents with ADRD, the VR advantages for psychological well-being and relationship quality were sustained at one month but not at three months. Parents with ADRD in the VR condition also showed significant improvement from baseline in social connection at the three-month follow-up, although change did not differ significantly between VR and VC. Social network support was also significantly higher than baseline among parents with ADRD at three months when collapsing across technology conditions. Among parents with MCI, the relative advantage of VC for relationship quality remained significant at the one-month follow-up but not at three months. The gradual attenuation of some parent-level benefits after the intervention ended is, in our view, consistent with the relational nature of the intervention rather than evidence against its effectiveness. Unlike interventions designed to build a lasting skill or habit, which persists without ongoing practice, the benefits of the Thrive intervention are rooted in shared family contact itself. It follows that improvements in well-being, relationship quality, and social connection would reasonably fade once that contact was no longer actively maintained. One practical implication is that technologies that make it easier for families to continue similar contact after a structured intervention period could help preserve gains longer term, an important direction for future research and program design.

The Thrive intervention also led to immediate meaningful personal and relational benefits for adult children, but unlike the pattern observed for parents, these effects did not differ by the type of technology used or by their parent’s cognitive status. Regardless of whether they used VR or VC, adult children reported significant improvements in psychological well-being, broader social connection, and relationship quality with their parents along with reductions in psychological distress, perceived stress, and caregiver guilt. Most of these gains (psychological well-being, relationship quality, perceived stress) were sustained through both follow-ups. The one notable exception was caregiver guilt: while both groups showed initial reductions, only adult children in the VR condition sustained this benefit at the one- and three-month follow-ups, whereas those in the VC condition showed no lasting change. One possible explanation is that the shared, immersive, and social nature of the VR sessions may create a more memorable sense of quality time (as suggested by the engagement findings, discussed below) that translates into longer-lasting relief from guilt over unmet caregiving expectations. This possibility was not directly tested here and merits dedicated investigation.

Turning to the secondary outcomes, analyses of self-reports and observational coding examined whether VR and VC elicited different forms of engagement among parents and adult children. Self-reports indicated that both VR and VC successfully fostered an equivalent sense of mutual relational connection. However, compared with VC, VR elicited a greater sense of sharing special moments, more learning, greater autobiographical reminiscing, less social strain among parents, and more positive emotion among adult children, regardless of the parent’s cognitive status. Observational coding provided converging evidence that VR fostered greater engagement. Although sessions in both conditions were similar in emotional warmth, VR elicited more emotional vitality among parents (laughter, happiness, wonder, and excitement) and more autobiographical reminiscing for both parents and adult children. Parents were also more likely than their adult children to lead conversations during VR sessions, whereas adult children tended to assume greater responsibility for generating conversation during VC. Together, these patterns suggest that Rendever’s VR platform may provide older adults with richer stimuli, including novel environments and memory cues, which help them initiate and sustain meaningful conversations with their adult children. More broadly, the findings underscore the value of examining multiple forms of engagement and using mixed methods (e.g., self-reports, human coding, automated coding) to understand the distinct affordances of different technologies. Consistent with prior research, the effectiveness of a technology likely depends on its specific purpose or function.^22,23,27^ The current findings suggest that Rendever’s socially enriched VR platform and VC serve distinct functions, with VR offering particular benefits for promoting older adults’ active engagement. Future research should also examine whether multi-user VR helps reduce caregiver burden by lessening the family member’s responsibility for initiating and sustaining conversation with their loved ones.

In addition, both observational and automated coding indicated that parents were more kinesically engaged during VR sessions than during VC. Although there was no significant moderation by parent’s cognitive status, parents with MCI showed greater kinesic engagement than parents with ADRD in the observational coding, but not in automated coding (see Extended Data Table 5). This pattern is partially consistent with our prior pilot study, in which automated coding showed greater kinesic engagement among parents with MCI than parents with ADRD during VR.^30^ Direct comparisons across studies should be made cautiously, as the pilot examined only VR, whereas the current trial compared engagement across both VR and VC and incorporated both automated and observational coding. The ways in which these technologies elicit different forms of engagement, as well as the potential mediating effects of engagement on personal and relational health, will be examined in a future manuscript.

Overall, both VR and VC significantly improved well-being and strengthened family connections for parents with cognitive impairments. However, VR (vs. VC) was especially effective in evoking shared memories and emotionally meaningful experiences, increasing parents’ active conversational and kinesic engagement, and enhancing psychological well-being. These findings support and extend existing research, which has focused primarily on solitary use of VR rather than as a social intervention for relationships. A recent review of 22 studies involving individuals with MCI and ADRD found that while VR was effective in enhancing positive emotions and autobiographical memories, it did not consistently improve overall quality of life.^52^ Notably, studies using fully immersive VR and content that recalled familiar, personally meaningful experiences showed stronger effects than those using less immersive VR or unfamiliar content. In contrast, another meta-analysis found conflicting results regarding the ability of VR and other digital technologies to induce engagement through reminiscence therapy.^53^ These discrepancies can likely be explained by a more detailed examination of the affordances of the technology.^54^ Rendever’s VR platform is specifically designed to stimulate human connection and mutual storytelling through interactive, emotional and cognitive engagement that is person-centered. Future analyses using the Thrive data will examine whether VR sessions that engaged autobiographical memory and reminiscence (Sessions 2 and 4) were more effective in improving older adults’ well-being than sessions that did not incorporate individualized memory-based content (Sessions 1 and 3).

The absence of intervention effects on loneliness was unexpected, particularly given that the trial was initiated during the latter stages of the COVID-19 pandemic, when social restrictions remained active in many communities. This finding may partly reflect that SLC residents had reliable access to peers and communal programming, potentially buffering against social isolation experienced by community-dwelling older adults. Indeed, baseline levels of loneliness were relatively low among parents in our sample. It is also important to consider the targeted nature of the intervention, which was designed specifically to foster social connection between older adults and distant family members. The intervention was also only four weeks long, which may have been too brief to shift overall loneliness. Despite the absence of effects for loneliness, the intervention did enhance relationship closeness and relationship quality with family members, with benefits for perceived social network support and social connection concentrated among parents with ADRD and, for social connection, specifically within the VR condition. Future research should test whether longer interventions, or delivery to older adults aging at home with less routine, in-person social contact, yield greater effects on loneliness.

Taken together, these findings suggest that socially enriched VR may be particularly effective for families coping with more advanced cognitive impairment, offering unique emotional and interactive benefits that support long-distance caregiving. Importantly, several of these positive effects persisted several months after the intervention concluded, a notable finding given the relatively short-term intervention and the fact that many of these effects included individuals living with dementia. These results highlight the potential of experiential, personalized, interactional and technology-based interventions to support the well-being of both older adults and their family members. While SLCs often focus on fostering relationships among residents – and prior research has demonstrated VR’s ability to enhance those community connections^9–13^ – this study draws attention to the value of sustaining intergenerational family bonds, despite geographic separation, specifically. Family relationships are distinct in their emotional significance, and engaging them meaningfully may offer therapeutic benefits for both older adults and their family members.

These findings have practical implications for SLCs and memory care programs. Given the differential patterns by cognitive status, activity directors and care teams might consider prioritizing immersive, socially-based VR family programming for residents with mild to moderate dementia, while offering VC as an effective, lower-cost alternative for residents with milder cognitive impairment. The high adherence and absence of serious adverse events observed here support the feasibility of integrating VR into routine programming, provided that training, screening, and safeguards are in place. Finally, the longer-term caregiver-guilt benefit in the VR condition suggests that family-oriented VR programming could be positioned not only as a resident well-being intervention but also as a support tool for the family members who care for them. Integrating adult children who are physically distant into their parent’s care could strengthen the family as a whole and encourage greater involvement in the SLC.

Given the sustained improvements associated with VR in the well-being of parents and adult children, a key area for future exploration is how this technology can be adapted for individuals aging in place, who face heightened risk of social isolation. Strengthening family relationships may be one of the most accessible and impactful avenues for promoting social health. We are currently collecting data using the same platform across the U.S., training in-home care aides and family care providers to use VR with individuals living with dementia. People without dementia should be able to use the system independently, with proper screening and safeguards. From a social impact perspective, community-dwelling older adults are also more likely to represent economically and ethnically diverse groups, and using VR in the home setting may provide much-needed respite for caregivers of individuals living with dementia. In this context, reducing caregiver burden might be an especially salient outcome to investigate. VR platforms are now widely regarded as safe and easy to operate, and activity directors, care providers, and older adults themselves can be readily trained in their use.

The theoretical and practical contributions of this intervention should be considered alongside its limitations. First, the trial compared two distinct approaches to technology-mediated social engagement rather than isolating the effect of VR itself. The Rendever VR platform combines immersive, structured activities with shared attention and cognitive stimulation, whereas VC primarily supports face-to-face conversation. Although comparing the platforms according to their intended uses enhances ecological validity, the study cannot determine which specific features of Rendever contributed to differences between conditions. Second, the physical presence of research staff during sessions could have increased parents’ positive affect and social engagement, although staff protocols were held constant across conditions. Third, because the trial did not include a no-treatment control group, improvements showing only a main effect of time cannot be attributed definitively to the intervention. Such changes may reflect components shared across conditions, including family interaction and staff attention, as well as regression to the mean or natural adjustment over time. Fourth, given the modest sample size, we did not adjust for multiple comparisons across outcomes, timepoints, and subgroups in order to preserve statistical power. This increased the risk of Type I error, particularly for three-way interactions near conventional significance thresholds, and these findings should therefore be viewed as preliminary. Fifth, despite recruitment efforts in low-income senior housing complexes and Latinx communities, the sample remained predominantly White and highly educated, which may limit generalizability. Nevertheless, sample characteristics align closely with the broader demographic profile of SLCs across the United States.^55^ Finally, because trial data were collected from September 2021 to March 2024, contextual effects related to the COVID-19 pandemic cannot be ruled out. However, data collection began after SLCs reopened to family visitors and vaccines were widely available. Although outbreaks occasionally delayed enrollment at new sites, data collection was not interrupted once it began within a community.

The results of the Thrive intervention suggest that both VR and VC are promising tools for supporting the well-being of older adults and their family members who are geographically separated. At the same time, social VR demonstrated distinct advantages, particularly for individuals with more advanced cognitive impairment and for sustaining reductions in caregiver guilt, which merit further exploration. These findings align with recent global calls to develop scalable, evidence-based solutions for social disconnection^56^, an urgent yet under-recognized threat to public health and societal well-being, as emphasized by the WHO Commission on Social Connection.^57^ Continued innovation will be essential to ensuring that social connection and well-being remain accessible to older adults and their families across diverse settings.

## Supplementary Material

Supplementary Files

This is a list of supplementary files associated with this preprint. Click to download.

• ThriveTrialnrreportingsummarycompletedPPT.pdf

• ThriveTrialNatureAgingExtendedDataFiguresandTables.pdf

• ThriveTrialCONSORT2025EditableChecklist.pdf

• TechnologyandFamilyThrivingStudyCTProtocolV1.5.docx1.pdf

• ThriveTrialSupplementalAppendixNatureAging.pdf

## Figures and Tables

**Table 1:** Baseline characteristics of parents and adult children in the overall sample and by technology condition

Baseline Characteristics	Overall Sample	Video Chat	Virtual Reality	Range
Parents	*n* = 186	*n* = 96	*n* = 90	
**Demographics**				
**Age, years, mean (SD)**	85.77 (7.50)	85.86 (6.98)	85.68 (8.06)	50 – 99
Sex, n (%)				–
Female	128 (68.8)	67 (69.8)	61 (67.8)	
Male	58 (31.2)	29 (30.2)	29 (32.2)	
Chronic health conditions, mean (SD)	3.99 (2.00)	3.99 (2.18)	4.00 (1.80)	1 – 11
MMSE score, mean (SD)	23.03 (3.72)	22.80 (3.95)	23.28 (3.45)	13 – 27
Cognitive status, n (%)				–
MCI	85 (45.7)	44 (45.8)	41 (45.6)	
ADRD	101 (54.3)	52 (54.2)	49 (54.4)	
Marital status, n (%)				–
Married	40 (21.5)	17 (17.7)	23 (25.6)	
Widowed	117 (62.9)	59 (61.5)	58 (64.4)	
Divorced	21 (11.3)	15a (15.6)	6b (6.7)	
Separated	2 (1.1)	0 (0)	2 (2.2)	
Never married	3 (1.6)	3 (3.1)	0 (0)	
Unknown (declined)	3 (1.6)	2 (2.1)	1 (1.1)	
Race/Ethnicity (NIH/OMB), n (%)				–
American Indian or Alaska Native	0 (0)	0 (0)	0 (0)	
Asian	1 (.5)	0 (0)	1 (1.1)	
Black or African American	1 (.5)	1 (1)	0 (0)	
Hawaiian or Pacific Islander	0 (0)	0 (0)	0 (0)	
Hispanic or Latino	5 (2.7)	2 (2.1)	3 (3.3)	
White	176 (94.6)	92 (95.8)	84 (93.3)	
More than one race	2 (1.1)	0 (0)	2 (2.2)	
Unknown or not reported	1 (.5)	1 (1)	0 (0)	
Education, n (%)				–
High school or less	19 (10.2)	8 (8.3)	11 (12.2)	
Some college/Vocational	44 (23.7)	22 (22.9)	22 (24.4)	
Bachelor’s degree	54 (29.0)	29 (30.2)	25 (27.8)	
Graduate/Advanced degree	62 (33.3)	34 (35.4)	28 (31.1)	
Unknown or not reported	7 (3.8)	3 (3.1)	4 (4.4)	
**Primary outcome variables, mean (SD)**	*n* = 168 – 180	*n* = 84 – 92	*n* = 84 – 92	**Scale Range**
*Psychological well-being* ^1^	−0.06 (0.91)	−0.07 (0.95)	−0.06 (0.87)	
Quality of life (QOL-AD)	2.95 (0.46)	2.94 (0.46)	2.96 (0.45)	1 – 4
Thriving	3.79 (0.61)	3.79 (0.64)	3.79 (0.58)	1 – 5
Positive affectivity	3.44 (0.84)	3.44 (0.85)	3.43 (0.83)	1 – 5
*Psychological distress* ^1^	0.07 (0.86)	0.07 (0.88)	0.06 (0.85)	
Depression (GDS)	2.73 (2.69)	2.68 (2.87)	2.79 (2.50)	0 – 15
Mental health symptoms (MHI5)	2.01 (0.74)	2.00 (0.72)	2.02 (0.77)	1 – 6
Negative affectivity	1.81 (0.69)	1.86 (0.70)	1.76 (0.67)	1 – 5
Loneliness (UCLA)	1.90 (0.73)	1.97 (0.73)	1.83 (0.73)	1 – 4
Social network support (LSN)	2.72 (1.01)	2.71 (1.03)	2.72 (1.01)	0 – 5
Social connection	48.68 (28.65)	47.60 (29.29)	49.77 (28.12)	−100 – +100
*Relationship quality with child* ^1^	−0.09 (0.89)	−0.12 (0.89)	−0.05 (0.90)	
Closeness	6.01 (0.99)	6.01 (0.93)	6.01 (1.05)	1 – 7
Relationship satisfaction	4.98 (1.15)	4.90 (1.18)	5.07 (1.12)	1 – 6
Communal coping	3.92 (0.80)	3.90 (0.81)	3.94 (0.80)	1 – 5
**Adult Children**	*n* = 186	*n* = 96	*n* = 90	
**Demographics**				
Age, mean (SD), years	56.38 (10.02)	56.72 (9.63)	56.03 (10.47)	25 – 76
Sex, n (%)				–
Male	75 (40.3)	35 (36.5)	40 (44.4)	
Female	111 (59.7)	61 (63.5)	50 (55.6)	
Relationship to parent, n (%)				–
Adult child	176 (94.6)	89 (92.7)	87 (96.7)	
Other (e.g., niece, grandchild)	10 (5.4)	7 (7.3)	3 (3.3)	
Marital status, n (%)				–
Married	137 (74.9)	74 (77.1)	63 (72.4)	
Widowed	2 (1.1)	0 (0)	2 (2.3)	
Divorced	19 (10.4)	9 (9.4)	10 (11.5)	
Separated	1 (0.5)	1 (1.0)	0 (0)	
Never married	24 (13.1)	12 (12.5)	12 (13.8)	
Race/Ethnicity (NIH/OMB), n (%)				–
American Indian or Alaska Native	0 (0)	0 (0)	0 (0)	
Asian	3 (1.6)	2 (2.1)	1 (1.1)	
Black or African American	1 (0.5)	1 (1)	0 (0)	
Hawaiian or Pacific Islander	1 (0.5)	1 (0)	0 (0)	
Hispanic or Latino	0 (0)	0 (0)	0 (0)	
White	177 (95.2)	90 (93.8)	87 (96.7)	
More than one race	2 (1.1)	1 (1)	1 (1)	
Unknown or not reported	2 (1.1)	1 (1)	1 (1)	
Education, n (%)				–
High school or less	9 (4.8)	4 (4.2)	5 (5.6)	
Some college/Vocational	20 (10.8)	11 (11.5)	9 (10.0)	
Bachelor’s degree	75 (40.3)	39 (40.6)	36 (40.0)	
Graduate/Advanced degree	81 (43.5)	42 (43.8)	39 (43.3)	
Unknown or not reported	1 (0.5)	0 (0.0)	1 (1.1)	
Employment status, n (%)				–
Full time	97 (52.7)	48 (50.5)	49 (55.1)	
Part time	23 (12.5)	13 (13.7)	10 (11.2)	
Not employed	59 (32.1)	32 (33.7)	27 (30.3)	
Other	5 (2.7)	2 (2.1)	3 (3.4)	
**Primary outcome variables, mean (SD)**	*n* = 179 – 181	*n* = 92 – 93	*n* = 87 – 88	**Scale Range**
*Psychological well-being* ^1^	−0.06 (0.92)	−0.14 (0.99)	0.02 (0.85)	
Thriving	4.10 (0.51)	4.10 (0.53)	4.11 (0.50)	1 – 5
Positive affectivity	3.57 (0.61)	3.49 (0.67)	3.66 (0.53)	1 – 5
*Psychological distress* ^1^	0.06 (0.85)	0.10 (0.93)	0.01 (0.77)	
Depression (CESD-10)	0.45 (0.40)	0.48 (0.45)	0.42 (0.35)	0 – 3
Mental health symptoms (MHI5)	2.03 (0.62)	2.06 (0.65)	2.00 (0.59)	1 – 6
Negative affectivity	1.74 (0.46)	1.76 (0.51)	1.72 (0.41)	1 – 5
Perceived stress	2.06 (0.63)	2.15 (0.64)a	1.96 (0.60)b	1 – 5
Caregiver guilt	2.25 (0.72)	2.23 (0.70)	2.28 (0.74)	1 – 5
Social connection	50.24 (32.59)	49.53 (34.88)	51.00 (30.17)	−100 – +100
*Relationship quality with parent* ^1^	−0.06 (0.88)	−0.09 (0.88)	−0.02 (0.89)	
Closeness	5.60 (1.20)	5.48 (1.33)	5.73 (1.05)	1 – 7
Relationship satisfaction	4.24 (1.23)	4.22 (1.19)	4.25 (1.27)	1 – 6
Communal coping	3.26 (0.95)	3.27 (0.90)	3.25 (1.00)	1 – 5

*Note.* MCI = Mild cognitive impairment. ADRD = Mild to moderate Alzheimer’s disease or related dementia. NIH/OMB = National Institutes of Health and Office of Management and Budget racial and ethnic categories prior to updated 2024 standards. GDS = Geriatric depression scale. UCLA = UCLA loneliness scale (adapted). LSN = Lubben social network. MHI5 = Mental health index (short form, 5-item). QOL-AD = Quality of life in Alzheimer’s disease scale. CESD = Center for Epidemiological Studies Depression inventory.

1 Psychological well-being, psychological distress, and relationship quality (for parents and adult children) are standardized composites. Sample sizes for the primary outcomes at baseline vary slightly due to a small number of dyads withdrawing after consent and to periodic missing data. Values in the same row with different subscripts are significantly different at *p* < .05, two-sided test of equality.

**Table 2:** *F*-Statistics and *p*-values from linear mixed models testing the effects of technology condition, cognitive status, and time (T1 to T2) on primary outcomes for parents

Parents Primary Outcome Variables	Main Effects			Interactions				Covariates		
	Time (T1 vs. T2)	Technology Condition (VC vs. VR)	Cognitive Status (MCI vs. ADRD)	Time × Technology	Time × Cog Status	Technology × Cog Status	Time × Technology × Cog Status	Age	Gender (M vs. F)	Chronic Health
	*F* *p*-value	*F* *p*-value	*F* *p*-value	*F* *p*-value	*F* *p*-value	*F* *p*-value	*F* *p*-value	*F* *p*-value	*F* *p*-value	*F* *p*-value
Psychological well-being	**3.71** ***p* = .056**	0.15 *p* = .697	8.90 *p* = .003	0.99 *p* = .321	**7.36** ***p* = .007**	2.64 *p* = .106	**6.15** ***p* = .014**	0.03 *p* = .868	1.40 *p* = .238	2.15 *p* = .144
Psychological distress	**5.00** ***p* = .027**	0.01 *p* = .936	1.69 *p* = .196	0.01 *p* = .924	0.06 *p* = .804	8.82 *p* = .003	0.04 *p* = .834	2.41 *p* = .123	0.35 *p* = .555	4.52 *p* = .035
Loneliness	0.59 *p* = .445	1.38 *p* = .242	0.03 *p* = .875	0.01 *p* = .930	1.07 *p* = .303	0.06 *p* = .811	0.002 *p* = .966	0.06 *p* = .814	1.39 *p* = .241	2.06 *p* = .153
Social network support^1^	0.61 *p* = .435	0.01 *p* = .930	6.91 *p* = .009	0.02 *p* = .893	**5.32** ***p* = .022**	0.11 *p* = .742	0.86 *p* = .356	0.01 *p* = .938	0.11 *p* = .741	0.27 *p* = .604
Social connection^2^	**5.52** ***p* = .020**	1.13 *p* = .290	1.96 *p* = .163	0.69 *p* = .408	0.21 *p* = .649	0.85 *p* = .359	**4.59** ***p* = .034**	1.69 *p* = .195	6.83 *p* = .010	3.07 *p* = .082
Relationship quality with child	**10.63** ***p* = .001**	0.34 *p* = .562	0.43 *p* = .514	0.02 *p* = .891	0.25 *p* = .620	0.19 *p* = .663	**4.04** ***p* = .046**	0.05 *p* = .828	0.32 *p* = .574	0.17 *p* = .679

*Note.* T1 = Baseline. T2 = Immediate post-intervention. VC = Video chat. VR = Virtual reality. MCI = Mild cognitive impairment. ADRD = mild to moderate Alzheimer’s disease or related dementia. M = Male. F = Female. *F*-tests for fixed effects from linear mixed models with restricted maximum likelihood estimation. Key effects relevant to hypothesis testing are shown in **bold**. Unless otherwise noted, *n* = 181.

1 *n* = 183.

2 *n* = 170.

See Table 3 for covariate-adjusted mean changes by technology condition and cognitive status, difference-in-differences contrasts, and corresponding effect sizes. See Table S5 for covariate-adjusted mean changes for the overall sample.

**Table 3:** Changes in parents’ primary outcomes from baseline to post-intervention (T1 to T2) by technology condition and cognitive status

Parents primary Outcome Variables	Within-Group Change: Baseline to Post-Intervention				Between-Group Difference in Change		
	Video Chat		Virtual Reality		Video Chat vs. Virtual Reality (DiD Contrast)		
	T2 - T1	*p*-value	T2 - T1	*p*-value	VR - VC	*p*-value	Effect Size
Psychological well-being							
MCI	.03 (−.14, .19)	.728	−.09 (−.26, .08)	.275	−.12 (−.36, .11)	.304	−.14 (−.39, .12)
ADRD	.05 (−.11, .21)	.545	.34 (.17, .50)	<.001	.29 (.06, .52)	.013	.32 (.07, .56)
Psychological distress							
MCI	−.10 (−.28, .09)	.297	−.09 (−.27, .10)	.370	.01 (−.25, .27)	.937	.01 (−.29, .31)
ADRD	−.10 (−.27, .07)	.256	−.13 (−.31, .05)	.164	−.03 (−.28, .22)	.825	−.03 (−.32, .25)
Loneliness							
MCI	−.08 (−.26, .09)	.357	−.08 (−.26, .10)	.398	.004 (−.25, .26)	.975	.01 (−.34, .35)
ADRD	.01 (−.16, .18)	.942	.02 (−.16, .19)	.843	.01 (−.23, .26)	.926	.02 (−.31, .35)
Social network support^1^							
MCI	−.05 (−.29, .20)	.716	−.14 (−.40, .11)	.272	−.10 (−.46, .26)	.588	−.10 (−.44, .25)
ADRD	.13 (−.11, .36)	.292	.26 (.02, .50)	.036	.13 (−.21, .47)	.441	.13 (−.20, .46)
Social connection^2^							
MCI	7.46 (0.44, 14.49)	.037	2.71 (−4.59, 10.01)	.465	−4.76 (−14.89, 5.37)	.355	−.17 (−.52, .18)
ADRD	−1.96 (−9.02, 5.11)	.585	8.82 (1.55, 16.09)	.018	10.77 (0.64, 20.91)	.037	.38 (.03, .73)
Relationship quality with child							
MCI	.20 (.03, .38)	.022	.04 (−.14, .22)	.668	−.16 (−.41, .09)	.196	−.18 (−.46, .09)
ADRD	.07 (−.10, .24)	.402	.26 (.09, .43)	.004	.19 (−.05, .43)	.124	.21 (−.06, .48)

*Note*. MCI = Mild cognitive impairment. ADRD = Mild to moderate Alzheimer’s disease or related dementia. DiD = Difference-in-differences contrast. Unless otherwise noted, *n* = 181.

1 *n* = 183.

2 *n* = 170.

Data are estimated mean differences (95% CIs) and p-values for fixed effects from linear mixed models (Technology Condition × Cognitive Status × Time) controlling for age, sex, and chronic health conditions. Positive mean differences indicate increases in scores from baseline (T1) to post-intervention (T2); negative values indicate decreases. Effect size was calculated by (VR - VC) / pooled standard deviation at baseline. See Table 2 for full model results, and Table S5 for mean changes in the overall sample.

**Table 4:** F-Statistics and p-values from linear mixed models testing the effects of technology condition, cognitive status, and time (T1 to T2) on primary outcomes for adult children

Adult Children Primary Outcome Variables	Main Effects			Interactions				Covariates	
	Time (T1 vs. T2)	Technology Condition (VC vs. VR)	Cognitive Status (parent) (MCI vs. ADRD)	Time × Technology	Time × Cog Status	Technology × Cog Status	Time × Technology × Cog Status	Age	Gender (M vs. F)
	*F* *p*-value	*F* *p*-value	*F* *p*-value	*F* *p*-value	*F* *p*-value	*F* *p*-value	*F* *p*-value	*F* *p*-value	*F* *p*-value
Psychological well-being	**7.33** ***p* = .008**	2.75 *p* = .099	1.04 *p* = .309	0.08 *p* = .772	2.50 *p* = .116	3.45 *p* = .065	1.94 *p* = .166	2.23 *p* = .137	0.41 *p* = .521
Psychological distress	**5.50** ***p* = .020**	2.07 *p* = .152	0.21 *p* = .651	1.11 *p* = .294	0.33 *p* = .567	0.33 *p* = .567	0.77 *p* = .381	3.02 *p* = .084	0.01 *p* = .941
Perceived stress	**7.63** ***p* = .006**	4.30 *p* = .040	0.49 *p* = .486	0.36 *p* = .547	1.41 *p* = .236	0.00 *p* = .992	0.55 *p* = .461	6.89 *p* = .009	0.22 *p* = .883
Caregiver guilt ^1^	**4.00** ***p* = .047**	0.69 *p* = .408	2.81 *p* = .095	1.13 *p* = .289	0.09 *p* = .763	4.59 *p* = .034	0.02 *p* = .888	5.08 *p* = .025	0.15 *p* = .703
Social connection ^2^	**5.74** ***p* = .018**	0.06 *p* = .808	3.32 *p* = .070	0.98 *p* = .323	0.09 *p* = .765	0.25 *p* = .616	0.09 *p* = .759	1.17 *p* = .282	3.76 *p* = .054
Relationship quality ^1^	**12.15** ***p* < .001**	0.07 *p* = .788	1.63 *p* = .203	2.40 *p* = .124	0.62 *p* = .433	0.00 *p* = .998	1.90 *p* = .171	0.13 *p* = .722	0.01 *p* = .934

*Note*. T1 = Baseline. T2 = Immediate post-intervention. VC = Video chat. VR = Virtual reality. MCI = Mild cognitive impairment. ADRD = mild to moderate Alzheimer’s disease or related dementia. M = Male. F = Female. *F*-tests for fixed effects from linear mixed models with restricted maximum likelihood estimation. Key effects relevant to hypothesis testing are shown in bold. Unless otherwise noted, *n* = 182.

1 *n* = 180.

See Table 5 for covariate-adjusted mean changes for the overall sample and by technology condition, difference-in-differences contrasts, and corresponding effect sizes.

**Table 5:** Changes in adult children’s primary outcomes from baseline to post-intervention (T1 to T2) in overall sample and by technology condition

Adult Children Primary Outcome Variables	Within-Group Change: Baseline to Post-Intervention						Between Group Difference in Change		
	Overall Sample		Video Chat		Virtual Reality		Video Chat vs. Virtual Reality (DiD Contrast)		
	T2 - T1	*p*-value	T2 - T1	*p*-value	T2 - T1	*p*-value	VR - VC	*p*-value	Effect Size
Psychological well-being	.13 (.03, .22)	**.008**	.11 (−.02, .24)	.084	.14 (.01, .27)	.038	.03 (−.16, .21)	.772	.03 (−.17, .23)
Psychological distress	−.13 (−.24, −.02)	**.020**	−.07 (−.22, .08)	.353	−.19 (−.34, −.03)	.019	−.12 (−.33, .10)	.294	−.14 (−.39, .12)
Perceived stress	−.14 (−.24, −.04)	**.006**	−.17 (−.31, −.03)	.016	−.11 (−.25, .04)	.137	.06 (−.14, .26)	.547	.10 (−.22, .41)
Caregiver guilt^1^	−.08 (−.16, −.001)	**.047**	−.12 (−.23, −.01)	.029	−.04 (−.15, .07)	.515	.08 (−.07, .24)	.289	.12 (−.10, .33)
Social connection^1^	4.66 (.82, 8.50)	**.018**	6.59 (1.24, 11.93)	.016	2.73 (−2.79, 8.24)	.330	−3.86 (−11.54, 3.82)	.323	−.12 (−.35, .12)
Relationship quality with parent^1^	.13 (.06, .21)	**<.001**	.19 (.09, .29)	<.001	.07 (−.03, .18)	.179	−.12 (−.27, .03)	.124	−.13 (−.30, .03)

*Note*. VR = Virtual reality. VC = Video chat. DiD = Difference-in-differences contrast. Unless otherwise noted, *n* = 182.

1 *n* = 180.

Data are estimated mean differences (95% CIs) and *p*-values for fixed effects from linear mixed models (Technology × Cognitive Status × Time) controlling for age and sex. Positive mean differences indicate increases in scores from baseline (T1) to post-intervention (T2); negative values indicate decreases. Effect size was calculated by (VR - VC) / pooled standard deviation at baseline. See Table 4 for full model results

**Table 6:** Differences in secondary outcomes by technology condition for parents and adult children

Secondary Outcome Variables	Parents				Adult Children			
	Video Chat	Virtual Reality	p-value	Effect Size	Video Chat	Virtual Reality	p-value	Effect Size
**Self-reported engagement**	*n* = 91	*n* = 82–84			*n* = 91	*n* = 84		
Positive emotions	4.03 (3.91, 4.14)	4.15 (4.03, 4.27)	.150	.22 (−.08, .52)	3.65 (3.52, 3.77)	3.89 (3.76, 4.02)	**.009**	.40 (.10, .70)
Negative emotions	1.22 (1.17, 1.26)	1.15 (1.10, 1.20)	.061	−.28 (−.58, .01)	1.19 (1.14, 1.24)	1.19 (1.13, 1.24)	.856	−.02 (−.32, .27)
Relational connection	4.48 (4.40, 4.55)	4.45 (4.37, 4.53)	.645	−.07 (−.37, .22)	4.23 (4.13, 4.33)	4.14 (4.03, 4.25)	.243	−.18 (−.48, .12)
Relational strain	1.46 (1.39, 1.52)	1.31 (1.24, 1.38)	**.006**	−.43 (−.73, −.13)	1.56 (1.47, 1.65)	1.51 (1.42, 1.60)	.471	−.11 (−.41, .19)
Learned something new	3.81 (3.68, 3.94)	4.20 (4.07, 4.33)	**<.001**	.65 (.34, .95)	3.41 (3.26, 3.56)	3.86 (3.70, 4.01)	**<.001**	.62 (.32, .93)
Reminiscence	3.57 (3.41, 3.73)	4.17 (4.01, 4.33)	**<.001**	.80 (.49, 1.11)	3.55 (3.40, 3.70)	3.90 (3.74, 4.05)	**.002**	.47 (.17, .78)
Shared special moments	4.28 (4.18, 4.37)	4.49 (4.39, 4.59)	**.003**	.46 (.15, .76)	4.02 (3.91, 4.14)	4.24 (4.11, 4.36)	**.013**	.38 (.08, .68)
**Observational- and computer-coded engagement**	*n* = 85	*n* = 78			*n* = 85	*n* = 78		
Conversational engagement	4.46 (4.34, 4.58)	4.43 (4.31, 4.56)	.771	−.05 (−.35, .26)	4.55 (4.45, 4.65)	4.46 (4.36, 4.57)	.235	−.19 (−.50, .12)
Conversational lead	2.46 (2.24, 2.68)	2.81 (2.58, 3.04)	**.034**	.33 (.03, .64)	2.99 (2.77, 3.20)	2.50 (2.28, 2.73)	**.002**	−.49 (−.80, −.17)
Reminiscence	3.01 (2.82, 3.20)	3.79 (3.59, 3.99)	**<.001**	.89 (.56, 1.21)	3.02 (2.82, 3.21)	3.76 (3.55, 3.96)	**<.001**	.82 (.50, 1.14)
Positive emotional engagement	4.29 (4.16, 4.42)	4.29 (4.16, 4.42)	.991	.00 (−.31, .31)	–	–	–	–
Active emotional engagement	3.69 (3.53, 3.86)	3.97 (3.80, 4.15)	**.023**	.36 (.05, .67)	–	–	–	–
Kinesic engagement (human-coded)	2.68 (2.50, 2.87)	3.12 (2.93, 3.30)	**.001**	.51 (.20, .82)	–	–	–	–
Kinesic engagement (computer-coded)^1^	0.033 (.030, .036)	0.039 (.036, .042)	**.009**	.41 (.11, .72)	–	–	–	–

*Note*.

1 For computer-coded kinesic engagement, Video Chat *n* = 87, Virtual Reality *n* = 80. Sample sizes for the secondary outcomes vary slightly due to a small number of dyad withdrawals, periodic missing data, recording errors, and if participants did not consent to video/audiotaping.

Tabled values are estimated means (95% CIs), *p*-values, and effect sizes (95% CIs), adjusted for covariates. Means reflect average ratings across the four technology sessions. For parents, *p*-values are based on linear ANCOVA models controlling for age, gender, chronic health conditions, and cognitive status (MCI vs. ADRD). For adult children, models controlled for age, gender, and parent’s cognitive status. None of the effects presented here were significantly moderated by the parent’s cognitive status. For full statistical results, see Extended Data Figure 9.

## Data Availability

Deidentified data, analysis code, and supporting documents will be made available via the NACDA-Open Aging Repository (NACDA-OAR) to researchers affiliated with institutions holding a Federal Wide Assurance (FWA). Access requires registration and agreement to NACDA’s conditions of use. For replication purposes, materials may also be requested directly from the corresponding author. The clinical trial protocol and statistical analysis plan can be accessed at clinicaltrials.gov (NCT05150990).

## Footnotes

1 Registration of this social-behavioral trial was completed in accordance with the NIH Policy on Dissemination of NIH-Funded Clinical Trial Information. Trial registration was submitted to ClinicalTrials.gov on October 28, 2021 and posted publicly on December 9, 2021.
